# Application of Interferon-γ Release Assay in the Assessment of T-Cell Immunity to SARS-CoV-2 Antigens in the Cohort of Pediatric Patients with Juvenile Idiopathic Arthritis

**DOI:** 10.3390/children11060736

**Published:** 2024-06-16

**Authors:** Katarzyna Kapten, Krzysztof Orczyk, Elzbieta Smolewska

**Affiliations:** 1 Department of Pediatric Cardiology and Rheumatology, Medical University of Lodz, 91-738 Lodz, Poland; katarzyna.kapten@umed.lodz.pl; 2 Department of Pediatric Infectious Diseases, Medical University of Lodz, 91-347 Lodz, Poland; krzysztof.orczyk@umed.lodz.pl

**Keywords:** JIA, SARS-CoV-2, COVID-19, cellular immunity, T-cells

## Abstract

**Background:** an accurate assessment of the immunity against SARS-CoV-2 can facilitate a better understanding and management of not only the recent coronavirus but similar pathogens as well. **Objective:** the aim of this study was to evaluate T-cell immunity with reference to antibody titers in a group of pediatric patients with autoimmune arthritides utilizing the widely known Interferon-γ Release Assay (IGRA). **Materials and Methods:** This study was conducted in the cohort of 55 children suffering from Juvenile Idiopathic Arthritis (JIA). This research analyzed the SARS-CoV-2 T-cell response measured by a specific quantitative IGRA, followed by a serological ELISA test measuring the presence and quantity of IgG, IgM, and IgA antibodies in serum. **Results:** The cellular response to SARS-CoV-2 measured by the IGRA test significantly correlated with the antibody titers, IgA (*p* < 0.00003, R = 0.537), IgG (*p* < 0.0001, R = 0.668), and IgG nucleocapsid protein (NCP) (*p* < 0.003, R = 0.0399), with no correlation with IgM levels. The antibody levels in patients receiving biological agents were significantly lower compared to the rest of the cohort (*p* = 0.0369), while traditional disease-modifying antirheumatic drugs had no such effect. **Limitations:** the main limitation of the research is the small sample size, mostly due to the specific cohort of patients and the lack of a healthy control. **Conclusions:** IGRA appears to be a viable tool in the accurate evaluation of T-cell responses to SARS-CoV-2, and serodiagnostics alone is not always sufficient in the assessment of immune responses.

## 1. Introduction

### 1.1. Current State of the Pandemic

Despite the substantially diminishing morbidity rate of SARS-CoV-2, with more than 700 million confirmed cases and almost 7 million fatalities worldwide in over 3 years, COVID-19 continues to be a global health concern [1]. As for today, scientists predict that it will remain an endemic issue for the foreseeable future [2]. In the course of the pandemic that was announced on 11 March 2020 by the World Health Organization, the scientists and healthcare professionals encountered a great many obstacles, showing the level of challenges that public health had to face [3]. While the rapid identification and isolation of infected individuals became the main objective at the beginning of the pandemic, currently, after most of the population was naturally exposed to the virus or/and vaccinated, the researchers’ attention shifted to accurately assessing one’s immunity which directly translates to the protection against SARS-CoV-2.

### 1.2. Humoral Immunity

Viruses such as SARS-CoV-2 initiate the infection with the viral antigen, activating adaptive immune responses through the antigen-presenting cells or B-cell receptors, inducing defense mechanisms against the pathogen. Following the infection, immunological memory is developed [4]. Due to the sterilizing qualities of antibodies, they were the first target for vaccine development and therefore the primary interest of the majority of the research. However, it was not long before it became clear that the antibody responses to COVID-19 were far more complex than simply marking the past infection. Early studies showed that higher antibody titers in SARS-CoV-2 infection are associated with more severe clinical manifestations of the disease [5,6], while a weak IgG response correlated with a significantly higher viral clearance, suggesting a pathological role of antibodies [7]. Interestingly, further research proved this correlation to be far more complex, and various factors, including the kinetics of seroconversion, antibody isotypes, and antigen specificity, should be considered to determine the effect of humoral response on disease severity. While a correlation between the longevity of antibody titers in serum and protection against re-infection was confirmed in numerous studies [8,9], factors like the severity of the infection or different variants of the virus may affect patients’ seropositivity [10,11].

### 1.3. Cellular Immunity

As adaptive immunity consists both of humoral and cellular components, the assessment of the T-cell response to COVID-19 appears to be no less relevant. The research on cellular immunity after SARS-CoV-1 infection indicated the high durability of T-cells, prevailing even 17 years after exposure to the virus, while a considerable drop in antibody titers was observed in the same patients just after 3–6 years [12,13]. Furthermore, it was noted that the cellular immunity gained by exposure to SARS-CoV-1 exhibited robust and expanding cross-reactivity to the N protein of SARS-CoV-2 and, even more interestingly, SARS-CoV-2-specific Interferon-γ (IFNγ) responses were found in donors previously unexposed to neither SARS-CoV-1 nor SARS-CoV-2 and thus are believed to come from exposure to unknown coronaviruses [13]. Similarly, the newest data on cellular memory against SARS-CoV-2 indicate a high durability of CD4+ and CD8+ T-cells [14], proposing its assessment, as they are essential for viral clearance and long-term, sustainable antiviral immunity. Moreover, the diagnostic T-cell assays for SARS-CoV-2 can be utilized in the cohorts of immunodeficient patients, who fail to produce antibodies or were given antibodies passively through immunoglobulins, but can still develop a cellular response to the virus [15,16]. While T-cells have a major protective role in the early stages of the disease, they can potentially contribute to the onset of fatal comorbidities, through T-cell exhaustion and hyperinflammation. As these new findings present a potential for new treatment options, further research on T-cell responses is essential [17,18].

### 1.4. Interferon-γ Release Assay (IGRA)

The Interferon-γ Release Assay (IGRA) is a widely known and validated test, utilized not only in the diagnostic process of diseases like tuberculosis [19] but also in other, mostly viral, infections [20,21]. Today, we can use the IGRA to determine the activity of SARS-CoV-2-reactive T-cells. Its high specificity, as well as sensitivity, has been confirmed in numerous research, emphasizing the potential in marking a long-lasting, durable cellular immunity, both shortly after exposure to the virus and weeks or months after the infection or vaccination against COVID-19 [22,23,24]. It becomes more apparent that reinfections with SARS-CoV-2 will remain a medical issue for quite some time. Therefore, the proper assessment of post-exposure immunity to SARS-CoV-2 can give us information on a person’s residual immunity and therefore the likelihood of a serious infection.

The objective of this study was to validate IGRA as a feasible tool to test cellular immunity, mediated by both CD4+ and CD8+ cells in possibly immunocompromised individuals exposed to SARS-CoV-2.

## 2. Materials and Methods

This prospective study was performed in the cohort of 55 pediatric patients diagnosed with Juvenile Idiopathic Arthritis (JIA) during their hospitalization in the Department of Pediatric Cardiology and Rheumatology, Medical University of Lodz. The recruitment for this study and all the laboratory testing that was part of this research took place between June 2021 and February 2023. The study group consisted of children between the ages of 2 and 16, in different stages of the disease, receiving various treatment regimes. The cohort included patients with both a negative (n = 47) and positive, PCR-confirmed, history of COVID-19 infection (n = 8), before this study commenced. Only 8 patients received the mRNA vaccine before being included in this study, while the rest of the group (n = 47) had not received the SARS-CoV-2 inoculation before this study commenced. None of the patients vaccinated against COVID-19 had a past history of SARS-CoV2 infection. The inclusion criteria for this study were confirmed JIA, in compliance with the International League of Associations for Rheumatology (ILAR) classification, and an age below 16 years old, with no lower age limit. All the patients in the cohort were previously immunized with the Bacillus Calmette–Guérin (BCG) vaccine against tuberculosis (TB), as it is an obligatory inoculation for newborns in Poland. None of the subjects reported having contact with TB. Additionally, all the patients who were qualified for biological treatment were tested for TB as part of the qualification procedure before receiving biological agents. The exclusion criteria for this study were a severe flare of JIA that required high immunosuppressive doses of steroids and the presence of concomitant autoimmune diseases, including diabetes mellitus, that could affect the results of this research.

SARS-CoV-2 T-cell response was measured using the EUROIMMUN Quan-T-Cell ELISA, catalog No. EQ 6841-9601 assay, which is a specific quantitative IGRA in whole blood. About 1.5 mL of blood was sampled during routine testing. The heparinized blood was then incubated in a set of three tubes (EUROIMMUN SARS-CoV-2 IGRA stimulation tube set, catalog No. ET 2606–3003): first tube—IGRA BLANK with no activating components, marking the individual’s Interferon as background; second tube—IGRA TUBE for specific T-cell stimulation by SARS-CoV-2 antigen spike protein; and third tube—IGRA STIM for unspecific T-cell stimulation with mitogen, for determining stimulation ability. Then, the obtained plasma was analyzed by the quantitative enzyme-linked immunosorbent assay (ELISA) to determine the concentration of released IFN-γ. All the patients were then tested for anti-SARS-CoV-2 antibodies, with EUROIMMUN Anty-SARS-CoV-2 ELISA, catalog No. EI 2606-9601 A (IgA); EUROIMMUN Anty-SARS-CoV-2 QuantiVac ELISA, catalog No. EI 2602-9601-10 G (IgG); and EUROIMMUN Anty-SARS-CoV-2-NCP, catalog No. EI 2606-9601-2 M (IgM). Additionally, the sensitive detection of IgG using the nucleocapsid protein (NCP) was used for the more accurate marking of antibodies [25] (EUROIMMUN Anty-SARS-CoV-2-NCP, catalog No. EI 2606-9601-2 G).

One of the prerogatives of this study was collecting blood samples needed for the tests mentioned above, during the standard blood drawing performed upon every hospitalization. Thus, in addition to the results of immune assays, the following parameters were analyzed as well: Total Blood Count, including leucocytes and platelets, as possible markers of ongoing inflammation and the most common inflammatory markers; C-reactive Protein (CRP); and Erythrocytes Sedimentation Rate (ESR).

All continuous variables were non-normally distributed based on the Shapiro–Wilk test. Therefore, all group comparisons were calculated utilizing the Mann–Whitney U test and Kruskal–Wallis H test. Spearman’s rank correlation coefficients were used to assess dependencies between quantitative variables. All statistical calculations were performed using Statistica 13.1 software (Statsoft Polska, Krakow, Poland). All the diagnostic tools were ordered from EUROIMMUN POLSKA, Wroclaw, Poland. This study was approved by the local Bioethics Committee, with approval number RNN/117/21/KE.

## 3. Results

The specifics of the cohort included in this study are presented in Table 1.

**Figure 1:**
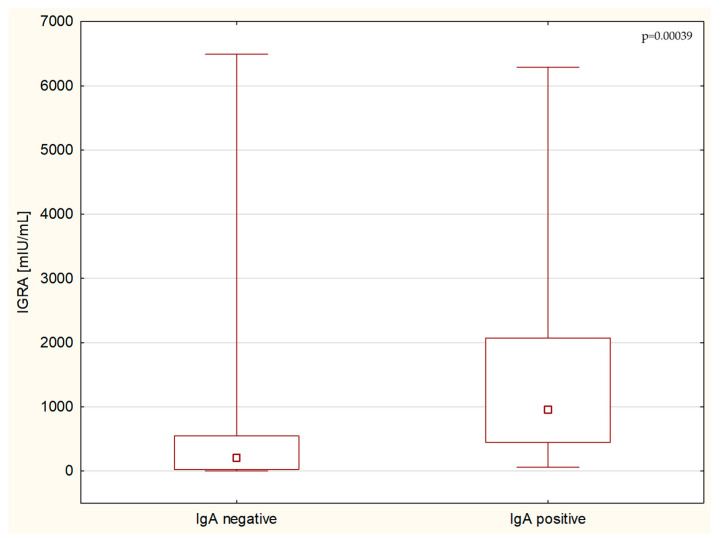
Group analysis of IGRA levels between IgA-negative and IgA-positive patients.

**Figure 2:**
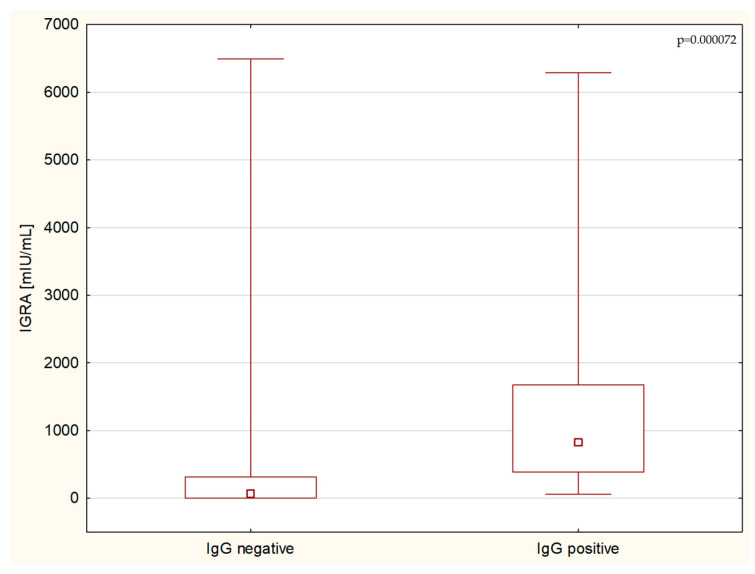
Group analysis of IGRA levels between IgG-negative and IgG-positive patients.

This research confirmed a significant correlation between the T-cell response to SARS-CoV-2 infection measured by the IGRA test and the humoral response measured by antibody titers. While IGRA positively correlated with the levels of IgA (*p* < 0.00003, R = 0.537) (Figure 1) and IgG antibodies, both in the test utilizing the modified S1 domain of the spike protein as an antigen (*p* < 0.0001, R = 0.668) (Figure 2), and in the highly sensitive NCP (*p* < 0.003, R = 0.0399), no correlation with IgM titers in serum was found.

Within the study group, five seronegative patients (in all antibodies classes), which constitute over 9% of the cohort, presented a SARS-CoV-2 T-cell response. While in patients with positive IgG titers, two had negative IGRA, and two had a cellular response in the grey area (100–200 mlU/mL), similarly, in patients with positive IgA titers, three had negative IGRA results. Regarding IgM response, one patient had positive IgM titers with no T-cell response; however, this was a patient with no IgG and no IgA response as well. This study analyzed the effects of different drug protocols on patients’ immunity. The investigated disease-modifying antirheumatic drugs (DMARDs) included methotrexate, sulfasalazine, cyclosporine, hydroxychloroquine, and azathioprine. None of these therapies significantly lowered immune responses, neither humoral nor cellular. Moreover, no correlation was found between children receiving glucocorticoids (GCs) in the study group and their immunity levels. However, it needs to be noted that none of the patients were on high doses of the GCs, none of the patients reached 2 mg of prednisone per kilogram of body weight, and none were receiving a prolonged GC therapy.

**Figure 3:**
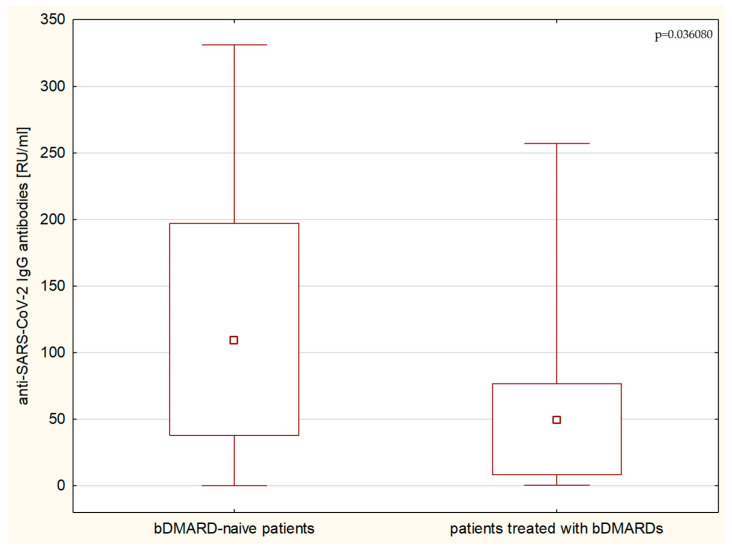
Group analysis of received biological treatment between IgG-negative and IgG-positive patients. bDMARDS—biological disease-modifying anti-rheumatic drugs.

Patients receiving biological treatment, including Tumor Necrosis Factor (TNF) blockers (adalimumab, etanercept), interleukin 6 inhibitor (tocilizumab), and Janus kinase inhibitor (baricitinib), had significantly lower (*p* = 0.0369) SARS-CoV-2 antibody titers when compared to the patients not receiving such treatment (Figure 3). The most promising results were noted with baricitinib; however, there was only one patient under this regime. Notably, no effects of biological agents were observed regarding cellular immunity, and no significant changes in T-cell levels of patients receiving such therapy were noted.

The age and sex of the patients did not correspond with immune responses. No correlation with standard blood tests was found, neither with inflammatory markers like CRP or ESR nor with leukocytosis or levels of platelets. Additionally, neither the SARS-CoV-2 antibody titers nor the specific T-cells levels correlated with the confirmed COVID-19 cases. However, it should be noted that only eight patients had tested positive for SARS-CoV-2 in Real-Time Polymerase Chain Reaction (RT-PCR) before this research commenced. Also, the history of COVID-like symptoms reported by the children’s parents did not correspond with the markers of the immune response.

## 4. Discussion

Cellular immunity is an invaluable component of the immune response to intracellular pathogens; thus, it is critical for the control of viruses, such as SARS-CoV-2 [17,26]. As randomized controlled trials have shown, the virus-specific cellular immune response prevents the spread of the virus within the host and eradicates the pathogen [27].

Evaluating cellular immunity as well as humoral responses to COVID-19 has proven to be an added value to standard serological testing [28]. A recent study confirmed a positive correlation between T-cell responses and SARS-CoV-2 antibody titers. These results are consistent with multiple studies, including one by Brand et al. [29] who evaluated T-cell responses against multiple SARS-CoV-2 structural proteins in a large number of patients, both exposed and unexposed to COVID-19, as well as with the results of Björkander et al. [30] who conducted a large population study on humoral and cellular immunity to SARS-CoV-2 in young adults. Moreover, Oja et al. postulated that antibody titers significantly correlate with S-specific CD4+ T-cell responses in patients with mild coronavirus disease, which remains in accordance with the results from the current study, as no patients within the cohort were reported to suffer from severe SARS-CoV-2 infection or required hospitalization [31]. In asymptomatic and mild cases of COVID-19, cellular immunity has been proven to be a better predictor of long-term immune memory than antibody titers, since T-cell responses remain at a detectable level when humoral memory starts to wane [32,33,34]. Interestingly, Wang et al. noted the importance of the coordination of cellular and humoral immunity for long-term protection, as he unraveled that high levels of virus-specific CD4+ T cells were associated with a slower decline in antibody titers [35]. However, the lack of consistent cutoff values for IGRA across different studies, with proposed values ranging from 25 IU/mL to 200 IU/mL, is notably a point that requires further validation. Applying universal values to this assay will benefit its utility both in clinical practice and research [22,23,24].

While the recent study proved the correlation between humoral and cellular responses, the lack of seroconversion in several patients who still mounted T-cell immunity should be noted. These results are concordant with multiple studies [36,37] postulating that exposure to SARS-CoV-2 can induce virus-specific T-cell responses without eliciting specific antibody production. In the study of Reynolds et al. on healthcare workers exposed to COVID-19, some seronegative subjects had undetectable T cell responses to spike protein, as well as T cells reactive to other SARS-CoV-2 antigens [38]. Thus, it can be speculated that pre-existing, cross-reactive memory T-cells can support the rapid clearance of infection in previously unexposed individuals [37,39].

**Figure 4:**
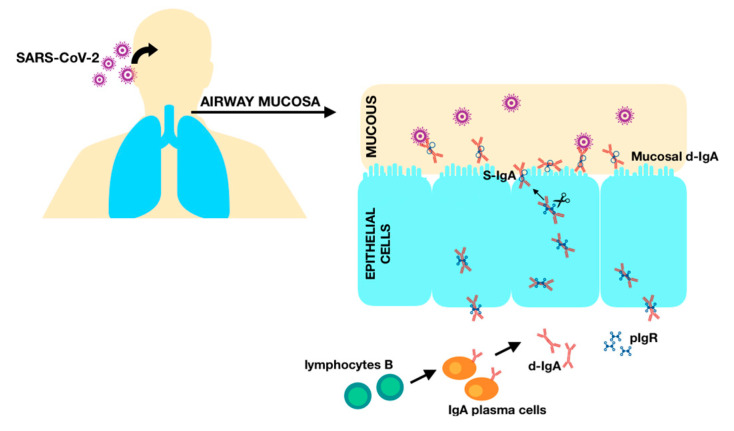
IgA eliminating SARS-CoV-2 on mucous membranes.

Positive IgA antibodies titers that were found in 33 out of 55 patients within the study group correlating with cellular responses to the virus support the previous findings, stating that an early SARS-CoV-2-specific humoral response is dominated by IgA antibodies and is crucial in virus neutralization [40,41]. IgA is produced by B lymphocytes with T-independent and T-dependent mechanisms and its subclass is the primary immunoglobulin in the respiratory tract, protecting the epithelial barriers from pathogens. While the IgA response to COVID-19 was proved to be highly effective and contributed significantly to virus neutralization, it is predominantly associated with early viral response as its titers in serum decreases notably as soon as a month after the infection, and while it has been found that specific local neutralizing IgA remains detectable in the saliva and airways for a longer period of time, it remains to be discovered whether these secretory antibodies may contribute to a long-term immunity against reinfection [40,41,42]. As SARS-CoV-2 is a mucosal-targeted virus (Figure 4), IgA plays a vital role in its management and should not be overlooked when assessing immunological responses in respiratory tract infections [43]. According to Infantile et al., the assessment of IgA antibodies in patients with early COVID-19 infection could help with closing the gap in serological testing, as they appear early, in high concentrations, and persist up to over 25 days from the disease onset [44]. However, there are some conflicting results regarding the correlation between the severity of COVID-19 and IgA levels. While Hennings et al. postulated that IgA-dominated responses was more likely to occur in asymptomatic infection with asymptomatic infection, Zervou et al. stated that high IgA titers positively correlated with the severity of the disease and were the highest in critically ill patients [42,45].

The figure illustrates the pathway of IgA from the blood circulation to mucous membranes in the human respiratory system. IgA is secreted by plasma cells, which are differentiated B cells after class switch recombination. IgA in secretions like mucus is found mainly in its dimeric form (d-IgA). Polymeric immunoglobulin receptor (pIgR) is a transmembrane protein that transports d-IgA across the mucosal epithelium. In order to cross the mucous layer by diffusion, the secretory component of pIgR (SC) undergoes endoproteolytic cleavage. Lastly, S-IgA consisting of d-IgA and SC can bind and neutralize pathogens, including SARS-CoV-2.

The data on immune responses to SARS-CoV-2 in patients suffering from immune-related rheumatic diseases (RD) are quite scarce; however, the available literature suggests that both cellular and humoral responses elicited in patients suffering from RD are comparable to healthy control groups and not impaired by standard immunomodulating therapies [46,47,48]. The recent study, for the most part, confirms these findings, where patients receiving DMARDs did not have impaired immunological mechanisms. However, the weakening of the humoral response was noted in patients receiving biological treatment. These results are consistent with the study conducted by Simon et al., who noted lower IgG titers, less frequent seroconversion, and reduced longevity of the humoral response in adult patients with RD receiving TNF-blockers and cytokine inhibitors [49]. Nonetheless, the data on clinical implications of the usage of biological agents in patients with RD suggest that the therapies should be continued; hence, they do not lead to more severe manifestations of COVID-19, including in the pediatric population [50,51]. In addition, many studies suggest that the usage of biological agents may have a protective effect against SARS-CoV-2 infection, since some of the drugs, like tocilizumab, have been successfully used in the management of COVID-19 [52,53,54,55]. Nevertheless, we should still be wary of rheumatic patients during this viral pandemic, as they may be at risk of poor outcomes of the infection; in particular, if the RD activity is high, they suffer from comorbidities or receive specific treatments like high doses of GCs or rituximab [55,56].

We acknowledge that our study has several limitations, such as a relatively small sample size and the lack of a control group. Adding a healthy control of children without RD may have yielded more solid results in the assessment of immunological responses to SARS-CoV-2 in patients with autoimmune disease when compared to their healthy peers. Additionally, due to a dynamic course of the pandemic, it has been nearly impossible to pinpoint the immunization stage of each and every patient from the onset of this study. Due to a low rate of PCR testing for SARS-CoV-2 within the pediatric population [57], including the cohort in the recent study, an undocumented or unrealized contact with the virus cannot be excluded. Moreover, a heterogeneous group with a relatively small number of patients being subjected to specific drug regimes allowed us to find trends rather than draw highly detailed conclusions regarding therapeutic recommendations. As the utilization of biological agents becomes more and more common in immune diseases, as well as in children, expanding the sample size to a larger cohort of pediatric patients receiving such treatment and focusing on specific agents may lead to the most reliable results and seems to be the best way forward for this research.

## 5. Conclusions

IGRA appears to be a valid tool in the assessment of individuals’ immunity to SARS-CoV-2 infection. T-cell responses proved to not only statistically correlate with the patient’s serological results, but in some cases indicate immunity in the absence of a humoral response. Thus, it is possible that, in some circumstances, marking solely the humoral responses to the virus can lead to an incomplete assessment of individual immunity and even negatively affect further clinical management. Looking into the future, there is a need for more comprehensive research to establish what level and type of residual immunity are needed to avoid severe disease after reinfection. Moreover, while, according to a recent study, the usage of DMARDs by patients with RD does not affect their immunity, a larger cohort study involving the pediatric population may help in investigating the effect of biological agents on humoral responses.

## Figures and Tables

**Table 1:** General characteristics of the study group.

	n = 55
**Male/Female**	14/41
**Age on examination (years)**	10.31 ± 4.16
**Positive IgA antibodies**	33
**Positive IgG antibodies**	40
**Positive IgG NCP antibodies**	23
**Positive IgM antibodies**	6
**Positive IGRA (>200 mlU/mL)**	41
**History of confirmed SARS-CoV-2 infection**	8
**Received SARS-CoV-2 vaccination**	8 *
**COVID-19-like symptoms in the history**	32
**Treatment protocol:**	
**Biological agents:**	22
**adalimumab**	13
**tocilizumab**	6
**etanercept**	2
**baricitinib**	1
**Methotrexate**	32
**Sulfasalazine**	8
**Hydroxychloroquine**	7
**Cyclosporine**	3
**Azathioprine**	1
**Glucocorticoids**	4

Values presented as mean ± standard deviation (SD). NCP—nucleocapsid protein; IGRA—Interferon-γ Release Assay. * None of the vaccinated subjects had a history of PCR-confirmed SARS-CoV-2 infection.

## Data Availability

The data used to support the findings of this study are included in the article. The supplementary data are available from the corresponding author upon request.
